# First Detection of an Alphaherpesvirus Gene in Humpback Whale Blow Samples Collected Noninvasively Using Unmanned Aerial Vehicles

**DOI:** 10.3390/v17111411

**Published:** 2025-10-23

**Authors:** Wataru Sekine, Junna Kawasaki, Kosuke Ohira, Kaixin Li, Misa Katayama, Ayano Ichikawa, Yuta Wakabayashi, Akiko Takenaka-Uema, Shin Murakami, Taisuke Horimoto

**Affiliations:** 1 Laboratory of Veterinary Microbiology, Graduate School of Agricultural and Life Sciences, The University of Tokyo, Tokyo 113-8657, Japan; w-sekine@g.ecc.u-tokyo.ac.jp (W.S.);; 2 Department of Infectious Disease Pathobiology, Graduate School of Medicine, Chiba University, Chiba 260-0856, Japan

**Keywords:** humpback whale, unmanned aerial vehicle, virome, herpesvirus

## Abstract

Viral infections have a significant impact on wildlife health, population dynamics, and ecosystem stability. Studies of cetaceans—key species in marine ecosystems—are challenging for viral infection research, owing to difficulties in collecting conventional biological samples. In this study, unmanned aerial vehicles (UAVs) were used in 2024 to noninvasively sample exhaled breath condensates (blows) from five groups of humpback whales (*Megaptera novaeangliae*) along the coastline of an island in the Pacific Ocean south of Japan. Comprehensive virome analysis revealed viral sequences related to 39 known virus species across 18 families, including nine that infect mammals. Notably, partial sequences of the *UL20* gene similar to an alphaherpesvirus previously identified in beluga whales were detected for the first time in the blows from these humpback whales. Our study demonstrates that UAV-based blow sampling is an effective tool for virological surveillance in cetaceans. Moreover, our findings aid in advancing our understanding of the diversity of viruses in marine mammals and supporting the development of noninvasive monitoring strategies that are critical for ensuring the conservation and health of these creatures.

## 1. Introduction

Cetaceans play critical roles not only in marine ecosystems but also in the broader global ecological balance. In recent years, efforts to mitigate global warming and rapid climate change have highlighted cetacean conservation as a simple, yet highly effective strategy, given that a single large whale can sequester approximately 33 tons of carbon dioxide (CO_2_) over its lifetime. After its death, its carcass sinks to the ocean floor, effectively locking away carbon for centuries. Thus, from a carbon sequestration perspective, cetaceans are essential allies for mitigating climate change [1]. Moreover, cetacean excreta provide vital nutrients to lower trophic organisms such as krill, thereby promoting primary ocean production, enhancing fishery resources, and supporting sustainable marine industries. Cetaceans also hold significant economic value through ecotourism, such as whale watching and dolphin shows. A single whale is estimated to generate a monetary value of approximately two million US dollars [1]. Thus, cetaceans are vital both ecologically and economically.

Understanding the ecological role of cetaceans is essential for ensuring their conservation. However, much about their biology remains unknown because they inhabit remote and often deep ocean areas. This is particularly true regarding the diseases they can be infected with, the data on which are limited. A study conducted in the Canary Islands of Spain found that approximately 80% of cetaceans examined between 2006 and 2012 had died as a result of several infectious bacterial, fungal, and viral diseases [2]. In another study investigating 89 stranded cetaceans along the coastline of Catalonia, Spain, between 2012 and 2019, 14.6% of deaths were found to be caused by cetacean morbilliviruses, 7.9% by *Brucella* spp., and 4.5% by *Crassicauda grampicola* [3].

Various cetacean species inhabit the Pacific Ocean areas near Japan. Strandings occur frequently along the Japanese coast, with multiple reports suggesting that viral infections may contribute to these events. For example, a mass mortality event of melon-headed whales in Hawaii in 2018 was attributed to an outbreak of cetacean morbillivirus [2], underscoring the potential of this pathogen to be a lethal threat to marine mammals worldwide [3,4,5,6]. Other viruses, including coronavirus in beluga whales (*Delphinapterus leucas*) and bottlenose dolphins [7,8], influenza virus in whales [9,10,11], and both the West Nile virus and polyomavirus in killer whales [12], have also been reported globally. Herpesviruses, which are capable of establishing latent infections in many vertebrates, including humans, have been detected in various cetacean tissues, such as the skin, lungs, and lymph nodes [13,14,15,16,17,18,19,20,21]. However, despite that herpesviruses have been documented in cetaceans from multiple regions, their diversity and virological characteristics remain poorly understood and warrant further investigation.

In recent years, noninvasive sampling methods using unmanned aerial vehicles (UAVs) have enabled the collection of cetacean exhaled breath condensates (blows), facilitating the analysis of host DNA and microbiomes in these marine mammals [22,23,24]. Notably, virome analysis of the blows is a promising method for detecting respiratory viruses, including those that were previously unknown. Indeed, six different viral genes have been identified in humpback whale (*Megaptera novaeangliae*) blows, demonstrating the usefulness of virome analysis [25]. All animals host microorganisms that significantly affect their physiology and immunity. The microbiome is closely connected to diet, disease, and human evolution, making its study essential to the life sciences. Similarly, virome analysis is a powerful tool for not only monitoring infectious diseases in cetaceans but also understanding host ecology and evolution [26,27,28,29].

Since 2018, humpback whale migrations have been observed around Hachijojima Island in Tokyo Prefecture, Japan, where individuals stay from December to April for breeding and calving [25,30,31]. Despite their seasonal presence, few studies have investigated viral infections in these whales, and no viruses have been detected directly from blows collected from whales in the coastal waters of Japan. Therefore, to contribute to the surveillance of viral infections in cetaceans, UAVs were used in this study to collect blow samples from humpback whales near Hachijojima Island for virome analysis in order to identify viruses potentially infecting these marine mammals.

## 2. Materials and Methods

### 2.1. Field Survey

**Figure 1:**
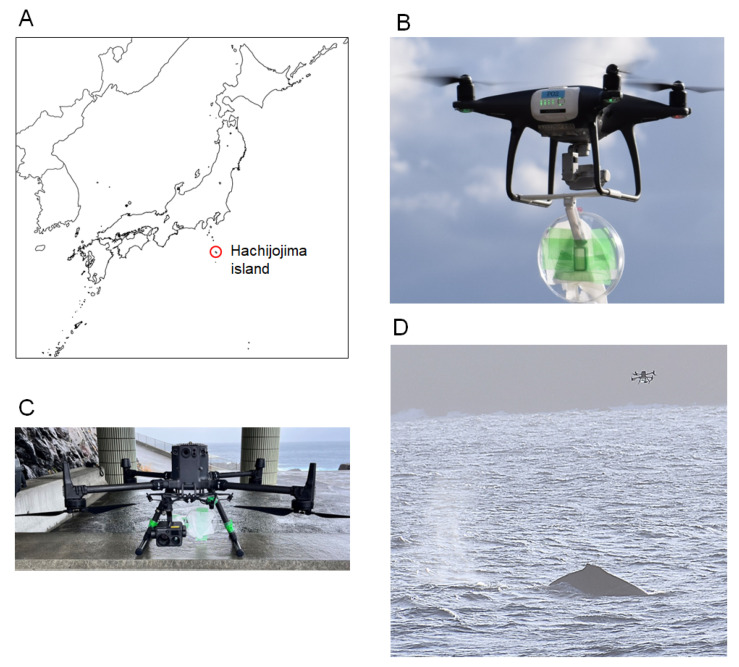
Sample collection. (**A**) Location of Hachijojima Island in Japan. (**B**,**C**) UAVs used for blow sampling: Phantom 4 Pro (**B**) and Matrice 300 RTK (**C**). Both UAVs were equipped with Petri dishes and absorbent papers for blow collection. (**D**) Attempting to collect blow samples from the whale using a UAV.

Sampling was carried out over 6 days (3 days each in February and March 2024) in the coastal waters off Hachijojima Island, Tokyo Prefecture, Japan (Figure 1A). Surveys were conducted under favorable weather conditions on both sunny and cloudy days with wind speeds below 10 m/s. Before the survey, the necessary permits were obtained from the Tokyo Metropolitan Port and Harbor Bureau and the Hachijo Town Office.

### 2.2. UAV Operation

We used Phantom 4 Pro (Figure 1B) and Matrice 300 RTK (M300RTK; Figure 1C) UAVs (SZ DJI Technology, Shenzhen, China) for blow sampling. Both UAVs were registered with the Civil Aviation Bureau of the Ministry of Land, Infrastructure, Transport, and Tourism (MLIT). All flights were operated by certified pilots (Kurikuri Craft Co., Ltd., Tokyo, Japan), with blanket flight permission and approval granted by the MLIT.

A 10 cm diameter Petri dish fitted with absorbent paper (Kimwipe; Kimberly-Clark, Dallas, TX, USA) was mounted on the UAV to collect whale blows. The UAV was flown at an altitude of approximately 2–5 m above the ocean surface and crossed directly through the whale blow to capture droplets. After each flight, the UAV was immediately retrieved, and both the Petri dish and camera lens were inspected for sample deposition.

### 2.3. Sample Processing

The papers with absorbed blow samples were soaked in Gene Keeper RNA & DNA Stabilization Solution (NIPPON GENE, Tokyo, Japan) and then manually squeezed to extract the absorbed fluid, which was collected using filter tips and stored. Samples were temporarily kept at 4 °C during transport to the laboratory and then stored at −80 °C until RNA extraction. Blow samples were pooled before RNA extraction to obtain sufficient RNA for sequencing. As environmental controls, seawater samples were collected near the blow collection site, supplemented with an equal volume of Gene Keeper solution, and stored at −80 °C until RNA extraction.

### 2.4. RNA Extraction, DNA Library Preparation, and Sequencing

The blow and seawater samples were centrifuged at 2300× *g* for 10 min at 4 °C. Subsequently, total RNA was extracted from the respective supernatants using the ISOGEN-LS reagent (NIPPON GENE) according to the manufacturer protocols. cDNA libraries were constructed using the Illumina Stranded mRNA Prep Ligation Kit (Illumina, San Diego, CA, USA) and sequenced on the NovaSeq 6000 platform (Illumina) with 150 bp paired-end reads.

### 2.5. Virome Analysis

Quality control of the paired-end RNA sequencing reads was performed using fastp (version 0.23.2), and nonviral sequences were removed by mapping analysis using HISAT2 (version 2.2.1) with a custom database based on RefSeq genomes, excluding viral genomes. The remaining reads were assembled de novo using rnaSPAdes, metaSPAdes (version 3.15.5), and MEGAHIT (version 1.2.9). The resulting contigs were subjected to BLASTx analysis using DIAMOND (version 2.1.8) to identify candidate viral genes. Additionally, BLASTn (version 2.2.26) was used to determine viral sequences that included noncoding regions. Contigs with a top BLAST hit to viral genomes and a length of 100 bp or more were considered virus-derived sequences and included in the downstream analyses.

### 2.6. Phylogenetic Analysis

Among the virus-derived sequences, those showing high similarity to sequences from viruses previously reported in cetaceans were selected with a sufficient read depth and contig length to evaluate their potential cetacean origin. To identify related alphaherpesviruses, *UL20* gene nucleotide sequences were aligned with those of other animal-sourced viruses using ClustalW. Using MEGA X software (v10.0.5), phylogenetic trees were constructed with the maximum-likelihood method under the Kimura 2-parameter model. For the *UL20* gene-encoded envelope protein, amino acid sequences were aligned using MUSCLE and analyzed using the maximum-likelihood method under the Le–Gascuel 2008 model. In total, 1000 bootstrap replicates were used to assess clade robustness.

### 2.7. Ethics Approval

The protocols and sampling procedures used in this study were approved by the Animal Experiment Committee of the University of Tokyo (Approval No. P23-174).

## 3. Results

### 3.1. Sample Collection

A geographical map of Hachijojima Island is shown in Figure 1A. To collect blow samples from humpback whales, we arranged UAVs with handmade blow collectors composed of plastic Petri dishes and absorbent paper (Figure 1B,C). After visually identifying the humpback whales, we estimated their likely surfacing positions and timing on the basis of their observed behavioral patterns. Through these observations, we predicted the movements of the whales and positioned the UAVs in areas where their reappearance was expected. Once the whales were visually confirmed again Via onboard cameras and visual sightings, the UAVs were flown approximately 2–3 m above the sea surface and maneuvered to track behind the individuals, attempting to intercept their blows directly (Figure 1D). Successful blow sampling was confirmed Via visual and infrared camera footage from the UAVs as well as by the presence of droplets adhering to the Petri dishes (Videos S1 and S2). During the 6-day sampling period, 33 UAV flights were conducted, and blow samples were successfully collected during five flights from five groups of humpback whales across two areas close to Hachijojima Island. Each blow sample contained exhalations from multiple individuals. At the time of sampling, no apparent abnormal behaviors or skin lesions were observed in any of the groups.

### 3.2. Virome Analysis

Total RNA was extracted from five pooled blow samples to improve the efficiency of viral sequence detection, as the RNA yield from each sample was below the required concentration for sequencing analysis. As controls, seawater samples were collected from two locations near the blow sampling sites and processed similarly. Total RNA extracted from the samples was used to prepare a cDNA library, and the cDNA was subjected to next-generation sequencing using the Illumina NovaSeq 6000 platform with 150 bp paired-end reads. This yielded 115,093,172 bp of sequence data from the blow samples and 51,824,166 bp from the seawater samples. As a preprocessing step before De Novo assembly, nonviral sequences were removed to reduce the risk of misassembly and the computational load. After filtering, the blow and seawater datasets contained 113,557,647 and 27,688,324 bp of sequence data, respectively. Contig construction was performed using the metagenomic assemblers SPAdes and MEGAHIT, resulting in 242,219 contigs from the blow sample and 1895 from the seawater sample.

**Figure 2:**
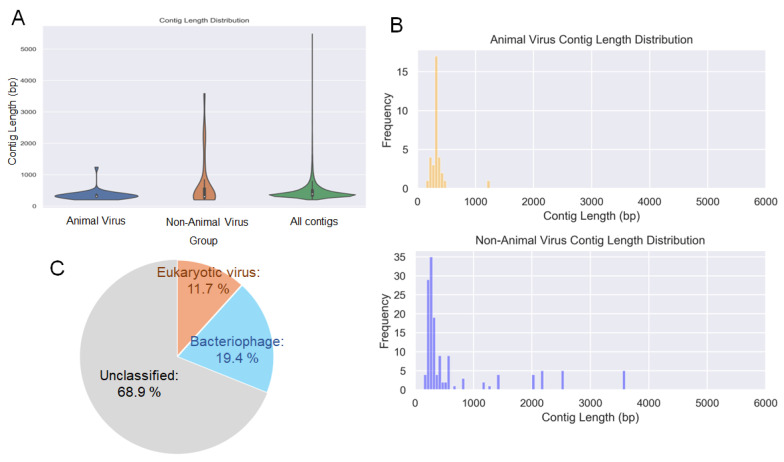
Virome analysis of the blow sample. (**A**) Length distribution of contigs containing viral genes classified as infecting animals, infecting non-animal hosts, and all contigs, including those that could not be classified. (**B**) Detailed length distribution of contigs containing viral genes infecting animals and those infecting non-animal species. (**C**) Taxonomic composition and relative abundance of viral contigs identified from the blow sample. Orange indicates eukaryotic viruses, light blue indicates bacteriophages, and gray indicates unclassified viruses.

To identify the viral genomes within these contigs, we conducted homology searches using DIAMOND BLASTx and NCBI BLASTn. The BLASTx search revealed 3209 and 11 contigs from the blow and seawater samples, respectively, with high sequence similarity to known viral genes, and further BLASTn filtering identified 655 viral contigs in the blow sample. No sequences similar to the viral genes were detected in the control seawater samples (Table S1). In this study, the median length of the assembled contigs was 386 bp, highlighting the inherent difficulty of assembling sequences from environmental samples. The viral contigs exhibited a similar pattern, with animal-associated viruses having a median length of 312 bp and a maximum length of 1242 bp (Figure 2A,B). Further analysis was restricted to contigs longer than 100 bp.

**Figure 3:**
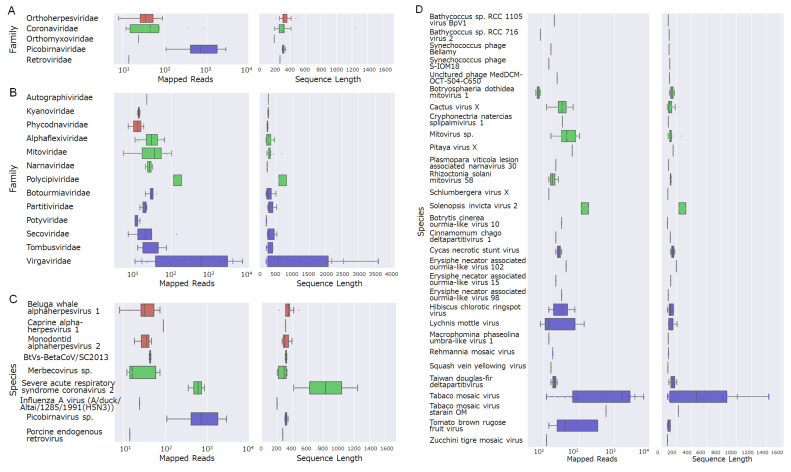
Boxplots summarizing sequences with high homology to viral genes from virus families that infect eukaryotes. The left panels show mapped read counts, and the right panels show sequence lengths: (**A**) virus genera that infect mammals, (**B**) virus genera that infect non-mammalian organisms, (**C**) virus species that infect mammals, and (**D**) virus species that infect non-mammalian organisms.

Next, we focused on viruses that could potentially infect cetaceans. To elucidate the overall taxonomic composition and relative abundance of viruses present in the collected samples, each viral sequence was assigned to a virus family on the basis of the lowest e-value obtained from the BLAST searches. The relative proportion of each family was then calculated on the basis of the number of sequence hits. According to the results, eukaryotic viruses accounted for 11.7%, bacteriophages for 19.4%, and unclassified viruses for 68.9% of all viral sequences (Figure 2C). Additionally, a total of 18 virus families were revealed from the analysis of contigs showing similarities to known viral genes. Among these, five families—*Orthoherpesviridae*, *Coronaviridae*, *Orthomyxoviridae*, *Picobirnaviridae*, and *Retroviridae*—have been reported to infect mammals, including cetaceans (Figure 3A). The remaining 13 families were classified as plant-infecting viruses or bacteriophages (Figure 3B). The virus species with the highest similarity are shown in Figure 3C: beluga whale alphaherpesvirus 1, caprine alphaherpesvirus 1, monodontid alphaherpesvirus 2, BtVs-BetaCoV/SC2013, merbecovirus, severe acute respiratory syndrome coronavirus 2, influenza A virus (A/duck/Altai/1285/1991(H5N3)), picobirnavirus, and porcine endogenous retrovirus. Thirty species classified as plant viruses or bacteriophages are shown in Figure 3D.

We applied additional filtering to identify cetacean-specific candidates among the mammalian viruses. Sequences of influenza virus, coronavirus, and picobirnavirus were excluded owing to their frequent detection in environmental sources such as wastewater, suggesting possible contamination [26,27,28,29,32,33]. Retroviral sequences were also excluded from further analysis because they were considered likely to have originated from endogenous retroviruses integrated into the host genome rather than reflecting active viral infections [34,35]. This screening revealed sequences that were highly similar to those of genes encoding herpesvirus capsid proteins, glycoprotein H, and the *UL20* protein (Supplementary Table S1). We focused on an approximately 500 bp sequence that exhibited notable similarity to the *UL20* gene of beluga whale alphaherpesvirus 1 (a known cetacean virus) and named it Megaptera novaeangliae-blow alphaherpesvirus-like sequence (Mn-blow αHV). We then conducted further phylogenetic analysis. The nucleotide sequence of Mn-blow αHV shared 75% similarity with a partial *UL20* gene sequence from beluga whale alphaherpesvirus 1, and the corresponding amino acid sequence showed 65% similarity to the envelope protein encoded by the same gene.

### 3.3. Phylogenetic Analysis

**Figure 4:**
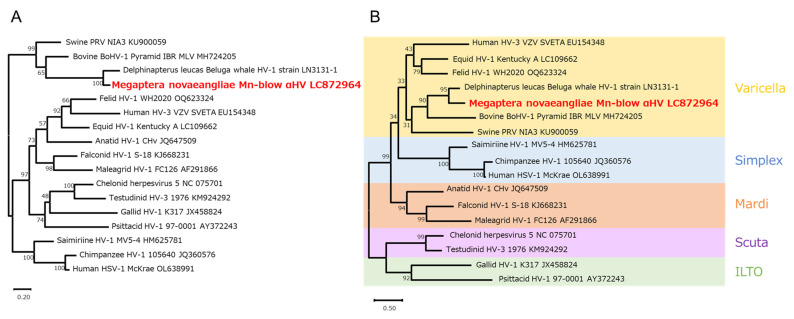
Phylogenetic trees of herpesviruses. Phylogenetic trees based on the *UL20* gene were constructed using the maximum-likelihood method: (**A**) nucleotide and (**B**) amino acid sequences of 17 virus species. The Kimura 2-parameter model was used for nucleotide sequences and the Le–Gascuel 2008 model for amino acid sequences. Bootstrap values (1000 replicates) are indicated on the branches. The scale bar shows the number of substitutions per site. The viruses are listed according to their strain name, accession number, and host origin. The Mn-blow αHV strain detected in this study is highlighted in red.

To determine the phylogenetic position of Mn-blow αHV, we performed phylogenetic analyses on the basis of the *UL20* gene nucleotide sequence and its corresponding amino acid sequence of the encoded envelope protein. Maximum-likelihood trees were constructed using 16 known alphaherpesvirus species. Mn-blow αHV clustered most closely with alphaherpesvirus 1, which was derived from the beluga whale (*Delphinapterus leucas*) [15]. Mn-blow αHV was also phylogenetically similar to viruses isolated from other artiodactyl mammals, such as cattle (BoHV-1) and pigs (PRV NIA3) (Figure 4A and Figure S1). In the amino acid-based phylogenetic tree, the virus species formed clades corresponding to the following known genera: *Varicellovirus*, *Simplexvirus*, *Mardivirus*, *Scutavirus*, and *Iltovirus*. Mn-blow αHV clustered within the Varicellovirus genus and formed a clade with beluga whale alphaherpesvirus 1, consistent with the results from the nucleotide-based analysis (Figure 4B and Figure S1).

## 4. Discussion

In this study, we successfully established a noninvasive UAV-based method for collecting blow samples from wild humpback whales. Although previous studies have reported similar sampling techniques, many relied on specialized modifications or expensive equipment [22,23,36]. Our approach makes use of a lightweight and low-cost sampling apparatus composed of absorbent paper attached to a Petri dish mounted on a stainless-steel plate, enabling the detection of viruses in whale blows. This technique significantly reduces operational barriers and is a practical sampling tool for marine mammal research.

The UAV used in this study had a lightweight design, allowing a flight time of approximately 40 min and multiple sampling attempts during a single flight. Additionally, numerous fully charged batteries ensured uninterrupted sampling throughout the 6-day field period, with no sampling sessions halted owing to battery depletion. However, because our sampling device lacks a sealing mechanism (e.g., a lid for the Petri dish), it may be more vulnerable to environmental contamination compared with closed systems. Future improvements should include the incorporation of a remotely controlled open–close mechanism while maintaining the light weight of the device [23].

Virome analysis of the whale blows revealed a diverse range of viral genes, encompassing 24 genera and 39 species of viruses. Among these genes, sequences highly similar to those of nine virus species from six genera known to infect mammals were identified. Notably, sequences resembling those of influenza virus, coronavirus, and picobirnavirus were detected. However, because these viruses are commonly found in environmental sources such as seawater and wastewater [26,27,28,29,32,33,37,38], their presence in the whale blows needs to be interpreted with caution and consideration of the possibility of cross-species transmission and interactions with environmental viruses. Although seawater samples were collected and analyzed as controls, the sequencing depth was insufficient for a robust comparison. When identifying novel viruses not yet registered in public databases, the local marine virome and surrounding environmental context must also be considered as references [39,40,41]. Additionally, the longest contig for the herpesvirus identified in this study was only 498 bp, which prevented reconstruction of the whole viral genome or a detailed genetic analysis. This limitation is likely due to sequence fragmentation and low viral nucleic acid concentrations, which are common challenges in environmental virome research. Moreover, to determine whether the detected sequences had originated from viruses infecting the whales or from environmental viruses, a thorough assessment of multiple factors such as read count, sequence length, and coverage must be assessed for accurate interpretation [42,43,44]. Although the sequences identified in this study are likely to be associated with whales, as based on their sequence similarities and the phylogenetic analysis, further research is needed to clarify their connection to the host.

Virome analysis of humpback whale blows has only been reported in one research article [36], making this present paper the second report. The authors of that other study targeted a population of humpback whales migrating in the Southern Hemisphere and identified sequences related to five virus genera. By contrast, we focused on North Pacific humpback whales and used a different analytical approach to detect different viruses, including herpesviruses. Therefore, this study represents the first reported detection of an alphaherpesvirus gene from humpback whale blows, demonstrating the effectiveness of noninvasive monitoring using exhaled breath samples.

Both alphaherpesviruses and gammaherpesviruses have been previously reported in various cetacean tissues, including the skin, lungs, liver, and genital mucosa [13,14,15,16,17,18,19,20,21]. Alphaherpesviruses are frequently detected in the nervous and respiratory systems. Their detection from the nasal mucosa of melon-headed whales and the lungs of false killer whales supports this tissue tropism [13]. The identified Mn-blow αHV in this study showed the highest sequence similarity (both in nucleotide and amino acid sequences) to beluga whale alphaherpesvirus 1 and was also phylogenetically related to herpesviruses isolated from cattle and pigs (Figure 4A,B). This suggests that this viral clade is highly conserved among the alphaherpesviruses isolated from *Cetartiodactyla*. Furthermore, based on the virological characteristics reported for cetacean alphaherpesviruses, Mn-blow αHV may either reside in or infect the respiratory tract of cetaceans.

The significance of this study extends beyond viral detection, as it also presents a novel monitoring strategy for detecting early signs of infectious disease outbreaks in wildlife. With increasing concerns over climate change and anthropogenic impacts on marine ecosystems, the need for noninvasive and rapid field sampling methods is paramount [45]. The UAV-based blow collection method demonstrated in this study is suitable not only for tracking specific pathogens but also for other applications. It can also be applied for the longitudinal monitoring of pathogen occurrence and genetic variation over time, laying the groundwork for the ecological surveillance of marine mammals. Moreover, as apex predators in aquatic ecosystems, cetaceans are often considered indicators of overall ecosystem health [22,23,46,47,48]. Our study findings, which include microbial data obtained from blow samples, can potentially contribute to the assessment of population health and the early detection of zoonotic disease risks. For populations that are difficult to observe or endangered, the importance of noninvasive approaches such as the one used in this study is expected to increase [49,50].

## 5. Conclusions

This study presents a new approach for investigating viral infections in cetaceans through blow sampling and expands the potential for monitoring respiratory and emerging infectious diseases in wild marine mammals. As viral pathogens that can cause respiratory illness, reproductive failure, and systemic infections [19,51], herpesviruses are of particular concern, and their noninvasive detection represents a significant advancement in the health management of marine mammals. Further refinement of UAV-based sampling techniques, comprehensive metagenomic analyses, and implementation of long-term monitoring will enhance our understanding of cetacean viral infections in changing marine environments.

## Data Availability

The sequence data of the Megaptera novaeangliae-blow alphaherpesvirus identified in this study have been deposited in the NCBI database (Accession No. LC872964). Additionally, the sequence data obtained using the NovaSeq 6000 platform (Illumina) with 150 bp paired-end reads have been submitted to the DDBJ Sequence Read Archive (DRA) under the accession numbers DRR704034 and DRR704035. All sequencing data used for the virome analysis are provided as Supplementary Materials (Table S1).

## Supplementary Materials

The following supporting information can be downloaded at: https://www.mdpi.com/article/10.3390/v17111411/s1, Figure S1: The Alignment of nucleotide (A) and amino acid (B) sequences of the *UL20* gene from 17 herpesvirus species; Table S1: Summary of viral contigs detected in humpback whale blow samples and seawater; Video S1: Collection of humpback whale blow using a UAV; Video S2: Infrared image of UAV collecting humpback whale blow.

## References

[1] Chami R., Cosimano T., Fullenkamp C., Oztosun S. (2019). Nature’s solution to climate change. Financ. Dev..

[2] West K.L., Silva-Krott I., Landrau-Giovannetti N., Rotstein D., Saliki J., Raverty S., Nielsen O., Popov V.L., Davis N., Walker W.A. (2021). Novel cetacean morbillivirus in a rare Fraser’s dolphin (*Lagenodelphis hosei*) stranding from Maui, Hawai’i. Sci. Rep..

[3] Domingo M. (1995). Evidence for chronic morbillivirus infection in the mediterranean striped dolphin (*Stenella coeruleoalba*). Vet. Microbiol..

[4] Sierra E., Fernández A., Zucca D., Câmara N., Felipe-Jiménez I., Suárez-Santana C., De Quirós Y., Díaz-Delgado J., Arbelo M. (2018). Morbillivirus infection in Risso’s dolphin grampus griseus: A phylogenetic and pathological study of cases from the Canary Islands. Dis. Aquat. Org..

[5] Costa-Silva S., Sacristán C., Duarte-Benvenuto A., Ewbank A.C., Soares R.M., Carvalho V.L., Castilho P.V., Cremer M.J., Vieira J.V., Lemos G.G. (2025). Morbillivirus and coronavirus survey in stranded cetaceans, Brazil. PLoS ONE.

[6] Vargas-Castro I., Peletto S., Mattioda V., Goria M., Serracca L., Varello K., Sánchez-Vizcaíno J.M., Puleio R., Nocera F.D., Lucifora G. (2023). Epidemiological and genetic analysis of cetacean morbillivirus circulating on the Italian coast between 2018 and 2021. Front. Vet. Sci..

[7] Mihindukulasuriya K.A., Wu G., St. Leger J., Nordhausen R.W., Wang D. (2008). Identification of a novel coronavirus from a beluga whale by using a panviral microarray. J. Virol..

[8] Wang L., Maddox C., Terio K., Lanka S., Fredrickson R., Novick B., Parry C., McClain A., Ross K. (2020). Detection and charac-terization of new coronavirus in bottlenose dolphin, United States, 2019. Emerg. Infect. Dis..

[9] Mandler J., Gorman O.T., Ludwig S., Schroeder E., Fitch W.M., Webster R.G., Scholtissek C. (1990). Derivation of the nucleoproteins (NP) of influenza A viruses isolated from marine mammals. Virology.

[10] Hinshaw V.S., Bean W.J., Geraci J., Fiorelli P., Early G., Webster R.G. (1986). Characterization of two influenza A viruses from a pilot whale. J. Virol..

[11] Lvov D.K., Zdanov V.M., Sazonov A.A., Braude N.A., Vladimirtceva E.A., Agafonova L.V., Oserovic A.M., Berzin A.A., Mjasnikova I.A., Podcernjaeva R.Y. (1978). Comparison of influenza viruses isolated from man and from whales. Bull. World Health Organ..

[12] St. Leger J. (2011). West Nile virus infection in killer whale, Texas, USA, 2007. Emerg. Infect. Dis..

[13] Miyoshi K., Nishida S., Sone E., Tajima Y., Makara M., Yoshioka M., Nakamura M., Yamada T.K., Koike H. (2011). Molecular identification of novel alpha- and gammaherpesviruses from cetaceans stranded on Japanese coasts. Zool. Sci..

[14] Noguchi K., Shimoda H., Terada Y., Shimojima M., Kohyama K., Inoshima Y., Maeda K. (2013). Isolation of a novel herpesvirus from a pacific white-sided dolphin. Arch. Virol..

[15] Davison A.J., Nielsen O., Subramaniam K., Jacob J.M., Romero C.H., Burek-Huntington K.A., Waltzek T.B. (2017). Genome sequence of an alphaherpesvirus from a beluga whale (*Delphinapterus leucas*). Genome Announc..

[16] Lee S.B., Lee K.L., Kim S.W., Jung W.J., Park D.S., Lee S., Giri S.S., Kim S.G., Jo S.J., Park J.H. (2024). Novel gammaherpesvirus infections in narrow-ridged finless porpoise (*Neophocaena Asiaeorientalis*) and false killer whales (*Pseudorca Crassidens*) in the Republic of Korea. Viruses.

[17] Pietroluongo G., Tucciarone C.M., Cecchinato M., Si H., Spadotto L., Danyer I.A., Isuru H., Wijesundera K., Ekanayake L., Centelleghe C. (2024). Coinfection with dolphin morbillivirus (DMV) and gammaherpesvirus in a spinner dolphin (*Stenella longirostris*) stranded in Sri Lanka. Viruses.

[18] Saliki J.T., Cooper E.J., Rotstein D.S., Caseltine S.L., Pabst D.A., McLellan W.A., Govett P., Harms C., Smolarek K.A., Romero C.H. (2006). A novel gammaherpesvirus associated with genital lesions in a Blainville’s beaked whale (*Mesoplodon densirostris*). J. Wildl. Dis..

[19] Sacristán C., Ewbank A.C., Duarte-Benvenuto A., Sacristán I., Zamana-Ramblas R., Costa-Silva S., Lanes Ribeiro V., Bertozzi C.P., Del Rio Do Valle R., Castilho P.V. (2024). Survey of selected viral agents (herpesvirus, adenovirus and hepatitis E virus) in liver and lung samples of cetaceans, Brazil. Sci. Rep..

[20] Vargas-Castro I., Crespo-Picazo J.L., Jiménez Martínez M.Á., Muñoz-Baquero M., Marco-Cabedo V., García-Párraga D., Sánchez-Vizcaíno J.M. (2024). Molecular detection of herpesvirus in a skin lesion of a humpback whale (*Megaptera novaeangliae*) from the western mediterranean sea. Eur. J. Wildl. Res..

[21] Arbelo M., Bellière E.N., Sierra E., Sacchinni S., Esperón F., Andrada M., Rivero M., Diaz-Delgado J., Fernández A. (2012). Herpes virus infection associated with interstitial nephritis in a beaked whale (*Mesoplodon densirostris*). BMC Vet. Res..

[22] Apprill A., Miller C.A., Moore M.J., Durban J.W., Fearnbach H., Barrett-Lennard L.G. (2017). Extensive core microbiome in drone-captured whale blow supports a framework for health monitoring. mSystems.

[23] Pirotta V., Smith A., Ostrowski M., Russell D., Jonsen I.D., Grech A., Harcourt R. (2017). An economical custom-built drone for assessing whale health. Front. Mar. Sci..

[24] Costa H., Rogan A., Zadra C., Larsen O., Rikardsen A., Waugh C. (2022). Blowing in the wind: Using a consumer drone for the collection of humpback whale (*Megaptera novaeangliae*) blow samples during the Arctic Polar nights. Drones.

[25] Kobayashi N., Kondo S., Tsujii K., Oki K., Hida M., Okabe H., Yoshikawa T., Ogawa R., Lee C., Higashi N. (2022). Interchanges and movements of humpback whales in Japanese waters: Okinawa, Ogasawara, Amami, and Hokkaido, using an automated matching system. PLoS ONE.

[26] Ahrens A.K., Selinka H.-C., Wylezich C., Wonnemann H., Sindt O., Hellmer H.H., Pfaff F., Höper D., Mettenleiter T.C., Beer M. (2023). Investigating environmental matrices for use in avian influenza virus surveillance—Surface water, sediments, and avian fecal samples. Microbiol. Spectr..

[27] Deboosere N., Horm S.V., Pinon A., Gachet J., Coldefy C., Buchy P., Vialette M. (2011). Development and validation of a concentration method for the detection of influenza A viruses from large volumes of surface water. Appl. Environ. Microbiol..

[28] Rönnqvist M., Ziegler T., Von Bonsdorff C.-H., Maunula L. (2012). Detection method for avian influenza viruses in water. Food Environ. Virol..

[29] Cuevas-Ferrando E., Pérez-Cataluña A., Allende A., Guix S., Randazzo W., Sánchez G. (2021). Recovering coronavirus from large volumes of water. Sci. Total Environ..

[30] Igarashi K., Tanabe A., Sahara H., Nozaki R., Kondo H., Katsumata T., Tamura S., Yamakoshi T., Mori M., Miyagi M. (2025). Application of DNA methylation–based age estimation to construct an age structure of humpback whales in a newly emerged wintering ground around Hachijojima Island, Tokyo metropolis, Japan. Ecol. Evol..

[31] Katsumata T., Hirose A., Nakajo K., Shibata C., Murata H., Yamakoshi T., Nakamura G., Kato H. (2021). Evidence of winter migration of humpback whales to the Hachijo Island, Izu archipelago off the southern coast of Tokyo, Japan. Cetacean Popul. Stud..

[32] Urayama S., Takaki Y., Nishi S., Yoshida-Takashima Y., Deguchi S., Takai K., Nunoura T. (2018). Unveiling the RNA virosphere associated with marine microorganisms. Mol. Ecol. Resour..

[33] Hamza I.A., Jurzik L., Überla K., Wilhelm M. (2011). Evaluation of pepper mild mottle virus, human picobirnavirus and torque teno virus as indicators of fecal contamination in river water. Water Res..

[34] Suntsova M., Garazha A., Ivanova A., Kaminsky D., Zhavoronkov A., Buzdin A. (2015). Molecular functions of human endogenous retroviruses in health and disease. Cell. Mol. Life Sci..

[35] Ruprecht K., Mayer J., Sauter M., Roemer K., Mueller-Lantzsch N. (2008). Endogenous retroviruses: Endogenous retroviruses and cancer. Cell. Mol. Life Sci..

[36] Geoghegan J.L., Pirotta V., Harvey E., Smith A., Buchmann J.P., Ostrowski M., Eden J.-S., Harcourt R., Holmes E.C. (2018). Virological sampling of inaccessible wildlife with drones. Viruses.

[37] Lodder W., De Roda Husman A.M. (2020). SARS-CoV-2 in wastewater: Potential health risk, but also data source. Lancet Gastroenterol. Hepatol..

[38] La Rosa G., Bonadonna L., Lucentini L., Kenmoe S., Suffredini E. (2020). Coronavirus in water environments: Occurrence, persistence and concentration methods—A scoping review. Water Res..

[39] Paez-Espino D., Eloe-Fadrosh E.A., Pavlopoulos G.A., Thomas A.D., Huntemann M., Mikhailova N., Rubin E., Ivanova N.N., Kyrpides N.C. (2016). Uncovering Earth’s virome. Nature.

[40] Kim Y., Van Bonn W., Aw T.G., Rose J.B. (2017). Aquarium viromes: Viromes of human-managed aquatic systems. Front. Microbiol..

[41] Simmonds P., Adams M.J., Benkő M., Breitbart M., Brister J.R., Carstens E.B., Davison A.J., Delwart E., Gorbalenya A.E., Harrach B. (2017). Virus taxonomy in the age of metagenomics. Nat. Rev. Microbiol..

[42] Geoghegan J.L., Holmes E.C. (2017). Predicting virus emergence amid evolutionary noise. Open Biol..

[43] Obbard D.J. (2018). Expansion of the metazoan virosphere: Progress, pitfalls, and prospects. Curr. Opin. Virol..

[44] Shi M., Lin X.-D., Tian J.-H., Chen L.-J., Chen X., Li C.-X., Qin X.-C., Li J., Cao J.-P., Eden J.-S. (2016). Redefining the invertebrate RNA virosphere. Nature.

[45] Goldbogen J.A., Friedlaender A.S., Calambokidis J., McKenna M.F., Simon M., Nowacek D.P. (2013). Integrative approaches to the study of baleen whale diving behavior, feeding performance, and foraging ecology. BioScience.

[46] Álvarez-González M., Suarez-Bregua P., Pierce G.J., Saavedra C. (2023). Unmanned aerial vehicles (UAVs) in marine mammal research: A review of current applications and challenges. Drones.

[47] De Oliveira L.L., Andriolo A., Cremer M.J., Zerbini A.N. (2023). Aerial photogrammetry techniques using drones to estimate morphometric measurements and body condition in South American small cetaceans. Mar. Mammal Sci..

[48] Irschick D.J., Martin J., Siebert U., Kristensen J.H., Madsen P.T., Christiansen F. (2021). Creation of accurate 3D models of harbor porpoises (*Phocoena phocoena*) using 3D photogrammetry. Mar. Mammal Sci..

[49] Afridi S., Laporte-Devylder L., Kline J.M., Penny S.G., Hlebowicz K., Cawthorne D., Lundquist U.P.S. (2025). Impact of drone disturbances on wildlife: A review. Drones.

[50] Schilling A.-K., Mazzamuto M.V., Romeo C. (2022). A review of non-invasive sampling in wildlife disease and health research: What’s new?. Animals.

[51] Melero M., Crespo-Picazo J.L., Rubio-Guerri C., García-Párraga D., Sánchez-Vizcaíno J.M. (2015). First molecular determination of herpesvirus from two mysticete species stranded in the Mediterranean sea. BMC Vet. Res..

