# Supplementary material for: First Detection of an Alphaherpesvirus Gene in Humpback Whale Blow Samples Collected Noninvasively Using Unmanned Aerial Vehicles
Source: Viruses. 2025 Oct 23;17(11):1411. doi: 10.3390/v17111411 (PMC12656974; doi:10.3390/v17111411)
Supplement: Supplementary file 1 [file viruses-17-01411-s001.zip › Figure S1.pdf]

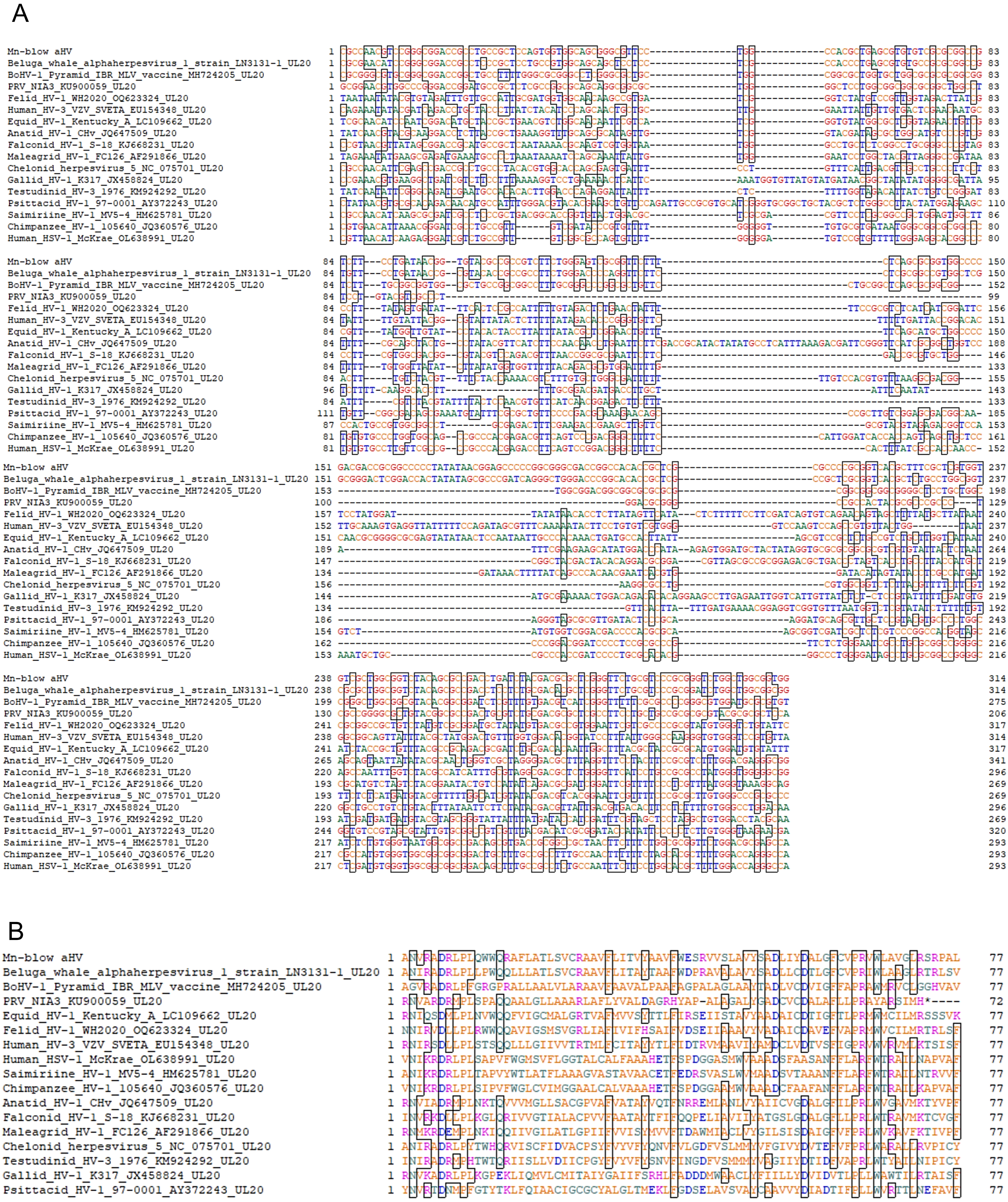

Figure S1
